# Chronic Condition Combinations and Health Care Expenditures and Out-of-Pocket Spending Burden Among Adults, Medical Expenditure Panel Survey, 2009 and 2011

**DOI:** 10.5888/pcd12.140388

**Published:** 2015-01-29

**Authors:** Abdulkarim M. Meraya, Amit D. Raval, Usha Sambamoorthi

**Affiliations:** Author Affiliations: Amit D. Raval, Usha Sambamoorthi, West Virginia University, Morgantown, West Virginia.

## Abstract

**Introduction:**

Little is known about how combinations of chronic conditions in adults affect total health care expenditures. Our objective was to estimate the annual average total expenditures and out-of-pocket spending burden among US adults by combinations of conditions.

**Methods:**

We conducted a cross-sectional study using 2009 and 2011 data from the Medical Expenditure Panel Survey. The sample consisted of 9,296 adults aged 21 years or older with at least 2 of the following 4 highly prevalent chronic conditions: arthritis, diabetes mellitus, heart disease, and hypertension. Unadjusted and adjusted regression techniques were used to examine the association between chronic condition combinations and log-transformed total expenditures. Logistic regressions were used to analyze the relationship between chronic condition combinations and high out-of-pocket spending burden.

**Results:**

Among adults with chronic conditions, adults with all 4 conditions had the highest average total expenditures ($20,016), whereas adults with diabetes/hypertension had the lowest annual total expenditures ($7,116). In adjusted models, adults with diabetes/hypertension and hypertension/arthritis had lower health care expenditures than adults with diabetes/heart disease (*P* < .001). In adjusted models, adults with all 4 conditions had higher expenditures compared with those with diabetes and heart disease. However, the difference was only marginally significant (*P* = .04).

**Conclusion:**

Among adults with arthritis, diabetes, heart disease, and hypertension, total health care expenditures differed by type of chronic condition combinations. For individuals with multiple chronic conditions, such as heart disease and diabetes, new models of care management are needed to reduce the cost burden on the payers.

## MEDSCAPE CME

Medscape, LLC is pleased to provide online continuing medical education (CME) for this journal article, allowing clinicians the opportunity to earn CME credit.

This activity has been planned and implemented in accordance with the Essential Areas and policies of the Accreditation Council for Continuing Medical Education through the joint sponsorship of Medscape, LLC and Preventing Chronic Disease. Medscape, LLC is accredited by the ACCME to provide continuing medical education for physicians.

Medscape, LLC designates this Journal-based CME activity for a maximum of 1 ***AMA PRA Category 1 Credit(s)™***. Physicians should claim only the credit commensurate with the extent of their participation in the activity.

All other clinicians completing this activity will be issued a certificate of participation. To participate in this journal CME activity: (1) review the learning objectives and author disclosures; (2) study the education content; (3) take the post-test with a 75% minimum passing score and complete the evaluation at www.medscape.org/journal/pcd; (4) view/print certificate.

**Release date: January 29, 2015; Expiration date: January 29, 2016**

### Learning Objectives

Upon completion of this activity, participants will be able to:

- Assess the overall burden of chronic disease among adults and its associated costs, according to a 2012 study
- Distinguish particularly expensive and inexpensive combinations of chronic disease in that study
- Evaluate the cost differential observed in the study when comparing adults with burdens of chronic disease
- Analyze out-of-pocket costs associated with different chronic diseases in the study

**EDITORS**

Camille Martin, Editor, *Preventing Chronic Disease*. Disclosure: Camille Martin has disclosed no relevant financial relationships.

**CME AUTHOR**

Charles P. Vega, MD, Clinical Professor of Family Medicine, University of California, Irvine. Disclosure: Charles P. Vega, MD, has disclosed the following relevant financial relationships: Served as an advisor or consultant for: McNeil Pharmaceuticals.

**AUTHORS AND CREDENTIALS**

Disclosures: Abdulkarim M. Meraya, MS has disclosed no relevant financial relationships. Amit D. Raval, MPharm, has disclosed the following relevant financial relationships: Received grants for clinical research from: Sanofi pharmaceuticals. Usha Sambamoorthi, PhD, has disclosed the following relevant financial relationships: Received grants for clinical research from: Sanofi pharmaceuticals.

Affiliations: Abdulkarim M. Meraya, Amit D. Raval, Usha Sambamoorthi, West Virginia University, Morgantown, West Virginia.

## Introduction

In the United States the average health care spending per person in 2012 was estimated to be $8,915, with $2.7 trillion total spent on health care (1). Most of these health care expenditures were associated with care for chronic conditions and associated risk behaviors (2). In 2012, 118 million (1 in 2) adults lived with at least 1 chronic condition from a list of 10 selected conditions, and among these adults 60 million lived with 2 or more chronic conditions (3).

However, little is known about how combinations of chronic conditions in adults affect total health care expenditures. Previous studies focused on the relationship between total health care expenditures and comorbidity indices (4–6) or number of chronic conditions (7–9) or how co-occurrence of 2 conditions affected health care expenditures (10–12). In 2009, average health care expenditures were $8,478 for adults with 2 or 3 chronic conditions and $16,257 for those with 4 or more chronic conditions (13).

Furthermore, out-of-pocket health care spending by individuals and families also impose an economic burden. In 2005, the average out-of-pocket spending for adults with 2 chronic conditions was $1,039, and for those with 3 or more chronic conditions, the average per-capita out-of-pocket spending was $1,865 (14).

The primary objective of this study was to assess the association between combinations of chronic conditions and total health care expenditures and out-of-pocket health care spending and burden. We selected arthritis, diabetes mellitus, heart disease, and hypertension for 3 reasons; first, these 4 diseases were among the most prevalent diseases among US adults (3); second, arthritis, heart disease, and diabetes mellitus were among the leading causes of death and disability (15); and third, the combinations of hypertension and heart disease, hypertension, and diabetes were most prevalent among the 2-condition combinations (16).

## Methods

### Study design

We used a cross-sectional design to examine the relationship between chronic condition combinations and total health care expenditures and out-of-pocket spending burden. We used household and medical conditions files of the Medical Expenditure Panel Survey (MEPS) for 2009 and 2011. Data from 2009 and 2011 were pooled to gain sample size. MEPS is a household survey of the noninstitutionalized civilian population and is conducted annually. The household component provides information on health status, demographic and socioeconomic characteristics, employment, access to care, and satisfaction with health care of the participants in the survey. Medical conditions files provide information on medical conditions reported by the households during participant interviews, during which the medical conditions described were recorded verbatim; these texts were translated into *International Classification of Diseases, 9th Edition, Clinical Modification* (ICD-9-CM) codes by professional coders (17). To preserve privacy, data provide nearly all of the diagnosis condition codes as 3-digit ICD-9 CM codes. In addition, MEPS data provide clinical classification codes, which are ICD-9-CM codes aggregated into clinically meaningful categories that group similar conditions (17).

The study sample consisted of adults aged 21 years or older who were alive during the calendar year and who had at least 2 of the following conditions: arthritis, diabetes mellitus, heart disease, and hypertension.

### Measures

#### Dependent variables

***Total health care expenditures:*** MEPS captured health care expenditures by collecting information on types of services and payers. Health care expenditures represented payments to health care providers (hospitals, physicians, and others). Type of services consisted of inpatient, outpatient, prescription drug, dental, vision, home health, and other. Other services included other medical equipment (expenditures for ambulance services, orthopedic items, hearing devices, prostheses, bathroom aids, medical equipment, disposable supplies, alterations and modifications, and other miscellaneous items or services that were obtained, purchased, or rented during the year). Type of payers included out-of pocket payments by patients or patients’ families, Medicare, Medicaid, private insurance, Veterans’ Administration, Tricare, and other federal and state payments. In MEPS, payments across types of services and payers for each person were summed to derive per-capita annual total health care expenditures.

***Out-of-pocket spending and burden***: Out-of-pocket spending consisted of self-reported payments for coinsurance and deductibles, as well as cash outlays for services, supplies, and other items not covered by health insurance (14,18). Out-of-pocket expenditures burden was calculated as the ratio of out-of-pocket spending to personal income (19) and expressed as a percentage, with out-of-pocket burden varying from 0 to 100. On the basis of the literature, we measured high out-of-pocket spending burden as spending 10% or more of personal income on health care (19).

#### Key Independent Variable

***Chronic condition combinations*:** Adults with at least 2 chronic conditions (arthritis, diabetes mellitus, heart disease, or hypertension) were classified into 11 mutually exclusive categories: 1) arthritis/diabetes; 2) arthritis/heart disease; 3) arthritis/hypertension; 4) diabetes/heart disease; 5) diabetes/hypertension; 6) heart disease/hypertension; 7) arthritis/diabetes/heart disease; 8) arthritis/diabetes/hypertension; 9) arthritis/heart disease/hypertension; 10) diabetes/heart disease/hypertension, and 11) arthritis/diabetes/heart disease/hypertension. We used diabetes/heart disease as the reference group in our analyses, because diabetes and heart disease were highly expensive conditions (20,21).

#### Other Independent Variables

Demographic variables included sex (male, female), age in years, race/ethnicity (white, African American, Latino, or other), and health insurance coverage (private, public, uninsured). Individuals were divided into 4 groups on the basis of their poverty status: poor (less than 100% federal poverty line), near poor (100% to less than 200%), middle income (200% to less than 400%), and high income (greater than or equal to 400%). Poverty status was calculated based on the family income in relation to the federal poverty line (based on family size and composition). Perceived health and mental status were measured with widely used validated scales. Perceived health status is the standard measured adopted by the SF-12 questionnaire. The MEPS queried respondents about “how one thinks of one’s health relative to the health of people in one’s age group” and scored responses on a 5-item scale: 1) excellent, 2) very good, 3) good, 4) fair, and 5) poor. Perceived mental health status was measured with a similar scale. A scoping review of self-reported mental health status measures indicated that a single-item mental health measure is associated with mental health and service use (22). The presence of other co-occurring physical conditions (asthma, cancer, chronic obstructive pulmonary disease, gastroesophageal reflux disease, thyroid disease, anxiety, and depression) was also included as an indicator variable.

### Statistical analysis

Health care expenditures were transformed on a natural logarithmic scale, and log-transformed expenditures were used as the dependent variable. To account for inflation, total expenditures were converted to 2011 dollars using the Consumer Price Index for medical services provided by the Bureau Of Labor Statistics (23). We used *t* tests to test differences in average health care expenditures by combinations of chronic conditions. Multivariable ordinary least squares (OLS) regressions were used to examine the association between combinations of chronic conditions and total health care expenditures after adjusting for sex, age, race/ethnicity, poverty status, type of insurance, perceived physical and mental health status, and number of chronic conditions other than arthritis, diabetes mellitus, heart disease and hypertension. For categorical variables, the percentage difference in expenditures was calculated by exponentiating the regression coefficient and subtracting 1 (ie, percentage difference = e^β^−1). In all regression models, diabetes/heart disease was used as the reference category.

Significant differences in high out-of-pocket spending burden by combination of chronic conditions were evaluated by χ^2^ tests. Multivariable logistic regressions were used to assess the relationship between chronic condition combinations and high out-of-pocket spending burden. In the adjusted models, we controlled for sex, race/ethnicity, age, poverty status, type of insurance, perceived physical health status, perceived mental health status, and number of co-occurring chronic conditions. In logistic regression models, diabetes/heart disease was used as the reference category. All analyses accounted for the complex survey design of MEPS and were conducted using SAS version 9.4 (SAS Institute, Inc). Because of multiple comparisons, variables with a *P* value of less than .01 were considered significant and those with a *P* value of less than .05 but greater than or equal to .01 were considered marginally significant.

## Results

### Description of the study sample

Our study sample consisted of 9,296 adults aged 21 years or older with at least 2 of the 4 chronic conditions (arthritis, diabetes mellitus, heart disease, and hypertension). Among adults with 2 conditions, the most prevalent combination was arthritis/hypertension (29.3%), followed by diabetes/hypertension (11.9%), heart disease/hypertension (11.1%), arthritis/heart disease (6.0%), arthritis/diabetes (3.8%), and diabetes/heart disease (1.0%). The most prevalent triad combination was arthritis/heart disease/hypertension (12.7%) followed by arthritis/diabetes/hypertension (11.0%), diabetes/heart disease/hypertension (4.8%), and arthritis/diabetes/heart disease (1.1%). Of adults in our study sample, 7.3% had all 4 conditions (arthritis/diabetes/heart disease/hypertension).

We found that a higher proportion of adults aged 21 to 64 years than those who were older had 2 chronic conditions, while a higher proportion of older adults (≥65 y) had 3 or more chronic conditions compared with those aged 21 to 64 years. There were significant differences in rates of specific combinations of chronic conditions by all the independent variables included in the study (Table 1).

**Table 1:** Study Sample Characteristics, by Chronic Condition Combination, Medical Expenditure Panel Survey, 2009 and 2011^a^

Characteristic	Chronic Condition Combination, Weighted %											*P* Value
	AR/DM	AR/HD	AR/HTN	DM/HD	DM/HTN	HD/HTN	AR/DM/HD	AR/DM/HTN	AR/HD/HTN	DM/HD/HTN	AR/DM/HD/HTN	
**All**	3.8	6.0	29.3	1.0	11.9	11.1	1.1	11.0	12.7	4.8	7.3	—
**Sex**												
Female	3.9	6.6	32.9	0.6	10.1	8.5	1.1	12.1	13.3	3.3	7.7	<.001
Male	3.6	5.2	25.0	1.5	14.0	14.2	1.2	9.7	12.0	6.7	7.0	
**Age, y**												
21–64	5.5	6.4	33.2	0.6	14.4	9.0	1.2	11.5	8.6	3.8	5.9	<.001
≥65	1.9	5.4	25.0	1.5	9.1	13.4	1.0	10.5	17.3	6.0	9.0	
**Race**												
White	3.7	6.7	30.1	1.0	9.3	12.3	1.2	9.8	14.3	4.6	6.9	<.001
Other	4.0	4.0	27.3	0.9	18.7	7.8	0.8	14.2	8.3	5.5	8.6	
**Poverty status**												
Poor	5.1	4.6	26.1	0.8	10.8	7.3	1.4	14.2	14.9	4.6	10.3	<.001
Near poor	3.1	5.1	30.3	0.8	10.3	10.2	1.2	10.7	14.3	4.6	9.5	
Middle income	4.0	6.2	27.8	0.9	12.9	10.9	1.2	10.8	12.2	5.6	7.5	
High income	3.4	6.8	31.3	1.3	12.5	13.2	0.9	10.1	11.3	4.4	4.8	
**Health insurance**												
Private	4.3	6.4	30.7	1.0	12.6	12.0	1.1	10.2	11.4	4.6	5.7	<.001
Public	2.8	5.6	26.0	1.2	9.4	10.0	1.2	11.8	15.9	5.4	10.7	
Uninsured	4.5	3.3	34.6	0.5^b^	19.2	7.2	0.7^b^	13.9	7.6	3.8	4.8	
**Perceived physical health**												
Excellent/very good	3.1	7.1	36.4	1.3	13.0	14.7	0.5	7.8	9.7	3.7	2.8	<.001
Good	4.4	4.9	28.0	0.9	13.8	10.4	1.1	12.1	13.3	5.0	6.2	
Fair/poor	3.8	6.0	24.0	0.9	8.6	8.4	1.7	12.8	14.9	5.8	13.1	
**Perceived mental health**												
Excellent/very good	3.3	6.0	31.2	1.3	13.5	12.9	0.8	9.8	10.7	5.1	5.4	<.001
Good	4.1	5.7	27.7	0.6	11.7	9.9	1.2	11.7	14.7	4.8	7.8	
Fair/poor	4.6	6.5	26.4	0.8	6.7	7.5	1.9	13.5	15.0	4.1	13.0	
**Other chronic conditions^c^**												
Yes	3.7	6.6	28.9	0.8	8.4	10.2	1.5	11.2	14.9	4.7	9.1	<.001
No	4.0	5..0	29.9	1.3	17.2	12.4	0.6	10.6	9.3	5.1	4.7	

Abbreviations: — , not applicable; AR, arthritis; DM, diabetes mellitus; HD, heart disease; HTN, hypertension.

a Based on 9,296 adults aged 21 years or older, alive during the calendar years 2009 and 2011, who reported having at least 2 of the following chronic conditions: arthritis, diabetes mellitus, heart disease, and hypertension.

b Unreliable estimates (cell count ≤5).

c Other chronic conditions included any of following chronic conditions: asthma, cancer, chronic obstructive pulmonary disease, gastresophageal reflux disease, thyroid disease, anxiety, or depression.

### Total health care expenditures

Adults with all 4 conditions had the highest average total health care expenditures ($20,016) (Table 2). Among adults with combinations of 2 or 3 chronic conditions, average health care expenditures differed by disease combination. Adults with arthritis/diabetes/heart disease had the highest average total health care expenditures ($19,109), followed by adults with arthritis/heart disease/hypertension ($16,275), diabetes/heart disease/hypertension ($15,528), arthritis/heart disease ($12,381), and diabetes/heart disease ($11,366). The lowest average total expenditures were observed among adults with diabetes/hypertension ($7,117) (Table 2).

**Table 2:** Total Health Care Expenditures (in 2011 US$), By Chronic Condition Combination, Medical Expenditure Panel Survey, 2009 and 2011^a^

Chronic Condition Combination	Total Health Care Expenditures, $		*P* Value
	Mean (SE)	95% CI	
All	11,466.0 (294.3)	10,886.1–12,046.6	—
AR/DM	11,126.8 (1,540.8)	8,088.5–14,165.0	.90
AR/HD	12,381.0 (1,343.1)	9,732.7–15,029.4	.57
AR/HTN	8,379.9 (365.4)	7,659.4–9,100.4	.03
DM/HD	11,366.2 (1,273.5)	8,855.0–13,877.4	1 [Reference]
DM/HTN	7,116.6 (379.4)	6,368.4–7,864.7	.002
HD/HTN	11,044.2 (732.2)	9,600.4–12,488.0	.82
AR/DM/HD	19,109.1 (2,860.3)	13,469.1–24,749.1	.01
AR/DM/HTN	10,622.2 (522.1)	9,592.7–11,651.8	.61
AR/HD/HTN	16,274.5 (1,481.8)	13,352.6–19,196.4	.007
DM/HD/HTN	15,528.3 (979.5)	13,596.9–17,459.8	.01
AR/DM/HD/HTN	20,015.6 (1,014.5)	18,015.3–22,016.0	<.001

Abbreviations: — , not applicable; AR, arthritis; CI, confidence interval; DM, diabetes mellitus; HD, heart disease; HTN, hypertension; SE, standard error.

a Based on 9,296 adults who were aged 21 years or older, alive during the calendar years, and reported having at least 2 of the following chronic conditions: arthritis, diabetes mellitus, heart disease, and hypertension.

Regression coefficient estimates, standard error, and percentage difference in total health care expenditures for combinations of chronic conditions compared with diabetes/heart disease are displayed in Table 3. Compared with adults with diabetes/heart disease, those with arthritis/hypertension and diabetes/hypertension had significantly lower total health care expenditures (44.3% and 40.5% respectively), after controlling for sex, race/ethnicity, age, poverty status, type of insurance, perceived physical and mental health status, and number of other chronic conditions. Adults with all 4 conditions had greater total health care expenditures (28.4%) than those with diabetes/heart disease. However, this difference was only marginally significant (*P* = .04)

**Table 3:** Parameter Estimates and Standard Errors of Chronic Condition Combinations From Ordinary Least Squares Regression on Log-Transformed Total Health Care Expenditures (in 2011 US$), Unadjusted and Adjusted Models, Medical Expenditure Panel Survey, 2009 and 2011^a^

Chronic Condition Combination	Unadjusted Model				Adjusted Model^b^			
	β	SE	% Difference (e^β^−1)	*P* Value	β	SE	% Difference (e^β^−1)	*P* Value
AR/DM	−0.346	0.154	−29.2	.03	−0.312	0.15	−26.8	.04
AR/HD	−0.131	0.132	−12.3	.32	−0.224	0.127	−20.1	.08
AR/HTN	−0.603	0.128	−45.2	<.001	−0.586	0.123	−44.3	<.001
DM/HD	1 [Reference]							
DM/HTN	−0.736	0.128	−52.1	<.001	−0.518	0.125	−40.5	<.001
HD/HTN	−0.267	0.124	−23.4	.03	−0.28	0.120	−24.4	.02
AR/DM/HD	0.414	0.180	51.3	.02	0.151	0.182	16.2	.41
AR/DM/HTN	−0.146	0.130	−13.6	.26	−0.189	0.125	−17.2	.13
AR/HD/HTN	0.098	0.126	10.3	.44	−0.106	0.119	−10.1	.37
DM/HD/HTN	0.216	0.138	24.1	.12	0.156	0.127	16.9	.22
AR/DM/HD/HTN	0.513	0.123	67.0	<.001	0.25	0.123	28.4	.04

Abbreviations: AR, arthritis; DM, diabetes mellitus; HD, heart disease; HTN, hypertension; SE, standard error.

a Based on 9,296 adults who were aged 21 years or older, alive during the calendar years, and reported having at least 2 of the following chronic conditions: arthritis, diabetes mellitus, heart disease, and hypertension.

b Adjusted model included sex, race/ethnicity, age, poverty status, type of insurance, perceived physical and mental health status, and number of co-occurring chronic conditions (reported having 1 of the following chronic conditions: asthma, cancer, chronic obstructive pulmonary disease, gastroesophageal reflux disease, thyroid disease, anxiety, depression). Percentage expenditures associated with chronic condition combinations were estimated by exponentiating the regression coefficients of dummy variables and subtracting 1 (ie, percentage difference = e^β^−1).

### Out-of-pocket health care spending

Adults with all 4 conditions had the highest average out-of-pocket spending ($1,814) (Table 4). Among adults with combinations of 2 or 3 conditions, adults with arthritis/heart disease/hypertension had the highest out-of-pocket spending ($1,760), followed by adults with arthritis/diabetes/heart disease ($1,740), diabetes/heart disease/hypertension ($1,585), arthritis/diabetes ($1,525), and diabetes/heart disease ($1,454). Adults with diabetes/hypertension had the lowest average out-of-pocket spending ($1,134) (Table 4). However, none of these differences were significant. Regression co-efficient estimates, standard error, and % difference in out-of-pocket spending for specific combinations of chronic conditions compared with diabetes/heart disease are displayed in Table 5. There were no significant differences in out-of-pocket spending by chronic condition combination, after controlling for sex, race/ethnicity, age, poverty status, type of insurance, perceived physical and mental health status, and number of other chronic conditions.

**Table 4:** Out-of-Pocket Health Care Expenditures and Burden (in 2011 US$), by Chronic Condition Combination, Medical Expenditure Panel Survey, 2009 and 2011^a^

Chronic Condition Combination	Out-of-Pocket Health Care Expenditures, $		*P* Value	Out-of-Pocket Health Care Expenditures Burden^b^		*P* Value
	Mean (SE)	95% CI		Mean (SE)	95% CI	
All	1,392.2 (35.5)	1,322.2–1,462.3	—	8.2 (0.2)	7.7–8.6	—
AR/DM	1,524.9 (198.7)	1,133.2–1,916.6	.80	8.4 (1.1)	6.2–10.6	.057
AR/HD	1,449.5 (109.4)	1,233.7–1,665.3	.98	7.3 (0.8)	5.7–8.9	.15
AR/HTN	1,226.6 (74.8)	1,079.1–1,374.0	.25	7.4 (0.4)	6.6–8.2	.07
DM/HD	1,454.4 (193.2)	1,073.4–1,835.4	1 [Reference]	5.7 (0.8)	4.2–7.2	1 [Reference]
DM/HTN	1,133.9 (49.0)	1,037.4–1,230.5	.12	5.7 (0.4)	4.9–6.5	.99
HD/HTN	1,305.9 (80.4)	1,147.3–1,464.4	.49	7.6 (0.8)	6.1–9.1	.09
AR/DM/HD	1,739.7 (251.4)	1,244.0–2,235.4	.38	9.6 (1.4)	6.7–12.4	.02
AR/DM/HTN	1,291.3 (50.7)	1,191.3–1,391.4	.42	9.7 (0.8)	8.2–11.3	<.001
AR/HD/HTN	1,759.9 (159.7)	1,444.9–2,074.9	.25	9.2 (0.6)	7.9–10.5	.001
DM/HD/HTN	1,585.4 (144.0)	1,301.6–1,869.3	.58	8.7 (0.8)	7.1–10.3	.01
AR/DM/HD/HTN	1,813.5 (110.1)	1,596.5–2,030.5	.10	12.2 (1.0)	10.2–14.2	<.001

Abbreviations: — , not applicable; AR, Arthritis; CI, confidence interval; DM: diabetes mellitus; HD, heart disease; HTN, hypertension; SE, standard error.

a Based on 9,296 adults who were aged 21 years or older, alive during the calendar years, and reported having at least 2 of the following chronic conditions: arthritis, diabetes mellitus, heart disease, and hypertension.

b Out-of-pocket expenditures burden was calculated as the ratio of out-of-pocket spending to personal income and expressed as a percentage, with out-of-pocket burden varying from 0 to 100. High out-of-pocket expenditures burden is the spending of 10% or more of personal income on health-related services.

**Table 5:** Parameter Estimates and Standard Errors of Chronic Condition Combinations From Ordinary Least Squares Regression on Log-Transformed Out-of-Pocket Expenditures (in 2011 US$), Medical Expenditure Panel Survey, 2009 and 2011^a^

Chronic Condition Combination	Log-Transformed Out-of-Pocket Expenditures^b^							
	β	SE	% Difference (e^β^−1)	*P* Value	β	SE	% Difference (e^β^−1)	*P* Value
	Unadjusted Model				Adjusted Model^c^			
AR/DM	−0.16	0.198	−14.8	.42	−0.061	0.2	−5.9	.76
AR/HD	−0.089	0.189	−8.5	.64	−0.168	0.19	−15.5	.37
AR/HTN	−0.331	0.173	−28.2	.057	−0.344	0.17	−29.1	.04
DM/HD	1 [Reference]							
DM/HTN	−0.328	0.185	−27.9	.08	−0.128	0.18	−12.0	.48
HD/HTN	−0.108	0.186	−10.2	.56	−0.177	0.18	−16.2	.33
AR/DM/HD	0.180	0.239	19.7	.45	0.110	0.22	11.6	.62
AR/DM/HTN	−0.131	0.185	−12.3	.48	−0.051	0.18	−5.0	.78
AR/HD/HTN	−0.032	0.182	−3.1	.86	−0.116	0.18	−11.0	.52
DM/HD/HTN	0.101	0.192	10.6	.60	0.161	0.19	17.5	.39
AR/DM/HD/HTN	0.173	0.189	18.9	.36	0.024	0.17	2.4	.20

Abbreviations: AR, Arthritis; CI, confidence interval; DM: diabetes mellitus; HD, heart disease; HTN, hypertension; SE, standard error.

a Based on 9,296 adults who were aged 21 years or older, alive during the calendar years, and reported having at least 2 of the following chronic conditions: arthritis, diabetes mellitus, heart disease, and hypertension.

b Out-of-pocket expenditures burden was calculated as the ratio of out-of-pocket spending to personal income and expressed as a percentage, with out-of-pocket burden varying from 0 to 100. High out-of-pocket expenditures burden is the spending of 10% or more of personal income on health-related services.

c Adjusted model included sex, race, age, poverty status, type of insurance, perceived physical and mental health status, and number of co-occurring chronic conditions (reported having 1 of the following chronic conditions: asthma, cancer, chronic obstructive pulmonary disease, gastroesophageal reflux disease, thyroid disease, anxiety, depression). Percentage expenditures associated with chronic condition combinations were estimated by exponentiating the regression coefficients of dummy variables and subtracting 1 (ie, percentage difference = e^β^−1.

### Out-of-pocket health care spending burden

Among all adults in our sample, 8.2% of income was spent as out-of-pocket on health care, and 17.1% of adults spent more than 10% of their income on health care (Table 6). Out-of-pocket spending burden varied by chronic condition combinations. In general, a higher proportion of people with 3 or 4 chronic conditions had high out-of-pocket spending burden compared with those with 2 chronic conditions.

**Table 6:** Unadjusted and Adjusted Odds Ratio and 95% Confidence Intervals for Chronic Condition Combinations From Logistic Regressions on High Out-of-Pocket Expenditures Burden^a^, Medical Expenditure Panel Survey, 2009 and 2011^b^

Chronic Condition Combination	Unadjusted OR			Adjusted^c^ OR		
	Weighted %	OR (95% CI)	*P* Value	Weighted %	OR (95% CI)	*P* Value
All	17.1	—	—	17.1	—	—
AR/DM	16.1	0.78 (0.45–1.37)	.40	16.1	0.75 (0.42–1.36)	.35
AR/HD	17.8	0.91 (0.53–1.55)	.72	17.8	0.77 (0.43–1.36)	.37
AR/HTN	14.8	0.7 (0.42–1.15)	.16	14.8	0.65 (0.39–1.11)	.12
DM/HD	14.4	1 [Reference]		14.4	1 [Reference]	
DM/HTN	11.8	0.61 (0.36–1.01)	.055	11.8	0.7 (0.41–1.20)	.20
HD/HTN	13.9	0.72 (0.43–1.21)	.22	13.9	0.68 (0.39–1.17)	.16
AR/DM/HD	23.7	1.86 (0.98–3.54)	.057	23.7	1.38 (0.70–2.74)	.36
AR/DM/HTN	18.8	1 (0.60–1.67)	.99	18.8	0.89 (0.52–1.53)	.67
AR/HD/HTN	21.9	1.12 (0.68–1.86)	.65	21.9	0.85 (0.50–1.46)	.57
DM/HD/HTN	20.9	1.1 (0.65–1.88)	.72	20.9	1.04 (0.59–1.83)	.88
AR/DM/HD/HTN	26.1	1.47 (0.88–2.45)	.14	26.1	1.12 (0.65–1.92)	.69

Abbreviations: — , not applicable; AR, Arthritis; CI, confidence interval; DM: diabetes mellitus; HD, heart disease; HTN, hypertension; OR, odds ratio.

a Out-of-pocket expenditures burden was calculated as the ratio of out-of-pocket spending to personal income and expressed as a percentage, with out-of-pocket burden varying from 0 to 100. High out-of-pocket expenditures burden is the spending of 10% or more of personal income on health-related services.

b Based on 9,296 adults aged 21 years or older, alive during the calendar years and reported having at least 2 of the following chronic conditions: arthritis, diabetes mellitus, heart disease and hypertension. High out-of-pocket spending burden as spending 10% or more of personal income on health care.

c Adjusted model included sex, race/ethnicity, age, poverty status, type of insurance, perceived physical and mental health status, and number of co-occurring chronic conditions (reported having one of the following chronic conditions: asthma, cancer, chronic obstructive pulmonary disease, gastresophageal reflux disease, thyroid disease, anxiety, depression). Percentage expenditures associated with chronic condition combinations were estimated by exponentiating the regression coefficients of dummy variables and subtracting 1 (ie, percentage difference = e^β^ − 1).

In multivariable logistic regression, after controlling for sex, race/ethnicity, age, poverty status, type of insurance, perceived physical and mental health status, and number of other chronic conditions, adults with all 4 chronic conditions were 47% more likely to have high out-of-pocket spending burden than adults with diabetes/heart disease. However, this difference was not significant (Table 6). Adults with diabetes/heart disease were more likely to use inpatient services compared with other groups (data not shown in the results and available on request).

## Discussion

We examined the relationship between combinations of chronic conditions and total health care expenditures, out-of-pocket spending on health care services, and out-of-pocket spending burden (defined as spending 10% or more of income on health care services). For this study, we selected adults with at least 2 conditions of 4 conditions — arthritis, diabetes mellitus, heart disease, and hypertension — which were chosen because they are highly prevalent or highly disabling or expensive. Without any adjustments, among adults with the selected conditions, those with all 4 conditions had the highest average total expenditures. Among adults with 2 or 3 chronic conditions, adults with arthritis/diabetes/heart disease had the highest total health care expenditures, followed by adults with arthritis/heart disease/hypertension, diabetes/heart disease/hypertension, arthritis/heart disease, and diabetes/heart disease.

After controlling for sex, age, race/ethnicity, poverty status, type of insurance, perceived physical and mental health, and presence of chronic conditions other than arthritis, diabetes, heart disease and hypertension, there were no differences in total health care expenditures among adults with 3 conditions when compared with those with diabetes/heart disease. Adults with arthritis/hypertension and diabetes/hypertension had lower total health care expenditures compared with those with diabetes/heart disease. These findings suggest that the association between chronic conditions and total health care expenditures vary by type of combination. Among adults with 2 or 3 of the 4 conditions, those with diabetes/heart disease may account for a high and disproportionate share of total health care expenditures.

It is well documented that diabetes and heart disease co-occur. Diabetes is a significant risk factor for incidence of heart disease (17). Our data suggested that adults with diabetes/heart disease were more likely to use inpatient services compared with other groups. Therefore, cost-saving efforts should focus on reducing hospitalizations for this group. It is also well- established that these 2 conditions are among expensive conditions treated in hospitals in the United States. For example, the estimated direct health care expenditures for diabetes were $176 billion in 2012 (18), and in 2010 the estimated direct and indirect expenditures for heart disease were $315.4 billion (19). Our study findings highlight the economic consequences of these 2 conditions when they co-occur. Future research and policy efforts should be directed toward multiple chronic conditions rather than having a single-disease focus.

In this study, out-of-pocket health care spending and burden did not differ by type of chronic condition combinations. Furthermore, there were no differences in out-of-pocket spending and burden for those with 3 or more conditions compared with those with diabetes/heart disease. This is in contrast to the findings from a study by Paez et al, which assessed the effect of number of chronic conditions on out-of pocket spending among adults with chronic conditions (14). However, Paez et al only compared number of chronic conditions rather than types of chronic conditions.

Our study had strengths and limitations. We used a nationally representative data of adults with chronic condition combinations and controlled for a comprehensive list of variables (sex, race/ethnicity, age, poverty status, type of insurance, perceived physical and mental health status, and number of co-occurring chronic conditions) which can affect health care expenditures. MEPS captured expenditures from all payer sources and types of services; MEPS data on medical conditions and health care use are self-reported, so they may be affected by recall bias. However, a study found that households accurately report chronic conditions that affect daily life (22). Our analysis was restricted to 4 chronic conditions, so our study findings are not generalizable to other conditions. Although we did not have information on the severity of the chronic conditions, which may affect the health care expenditures, we controlled for perceived physical and mental health status.

To the best of our knowledge, this study is the first to use MEPS data to analyze the relationship between combinations of chronic conditions and total health care expenditures and out-of-pocket spending and burden among adults with at least 2 of the studied conditions (arthritis, diabetes mellitus, heart disease, and hypertension). By restricting the analysis to adults with at least 2 chronic conditions, we found that type of chronic condition does affect average health care expenditures. We also found that total health care expenditures for people with 3 conditions were comparable to those of people with only diabetes and heart disease. Health care providers and payers should pay special attention to managing these 2 conditions to reduce total health care expenditures. However, out-of-pocket spending and burden did not differ by combination, suggesting that the burden on families and individuals may be the same when an individual has 2 or more of the chronic conditions included in our study.

## Figures and Tables

**Table:** 

**1. The activity supported the learning objectives.**				
**Strongly Disagree**				**Strongly Agree**
1	2	3	4	5
**2. The material was organized clearly for learning to occur.**				
**Strongly Disagree**				**Strongly Agree**
1	2	3	4	5
**3. The content learned from this activity will impact my practice.**				
**Strongly Disagree**				**Strongly Agree**
1	2	3	4	5
**4. The activity was presented objectively and free of commercial bias.**				
**Strongly Disagree**				**Strongly Agree**
1	2	3	4	5

## Post-Test Information

To obtain credit, you should first read the journal article. After reading the article, you should be able to answer the following, related, multiple-choice questions. To complete the questions (with a minimum 75% passing score) and earn continuing medical education (CME) credit, please go to http://www.medscape.org/journal/pcd. Credit cannot be obtained for tests completed on paper, although you may use the worksheet below to keep a record of your answers. You must be a registered user on Medscape.org. If you are not registered on Medscape.org, please click on the "Register" link on the right hand side of the website to register. Only one answer is correct for each question. Once you successfully answer all post-test questions you will be able to view and/or print your certificate. For questions regarding the content of this activity, contact the accredited provider, CME@medscape.net. For technical assistance, contact CME@webmd.net. American Medical Association's Physician's Recognition Award (AMA PRA) credits are accepted in the US as evidence of participation in CME activities. For further information on this award, please refer to http://www.ama-assn.org/ama/pub/about-ama/awards/ama-physicians-recognition-award.page. The AMA has determined that physicians not licensed in the US who participate in this CME activity are eligible for ***AMA PRA Category 1 Credits***™. Through agreements that the AMA has made with agencies in some countries, AMA PRA credit may be acceptable as evidence of participation in CME activities. If you are not licensed in the US, please complete the questions online, print the AMA PRA CME credit certificate and present it to your national medical association for review.

## Post-Test Questions

### Article Title: Chronic Condition Combinations and Health Care Expenditures and Out-of-Pocket Spending Burden Among Adults, Medical Expenditure Panel Survey, 2009 and 2011

**CME Questions**

1. You are seeing a 67-year-old woman with a history of type 2 diabetes, hypertension, and osteoarthritis. She recently retired as a principal of a local elementary school, and she is worried about the costs of care for her chronic medical conditions. What should you consider in general regarding the burden of chronic illness among US adults and its associated costs?
  - The average annual cost of health care for each US adult is approximately $30,000
  - Half of US adults have a common chronic illness
  - Previous research has failed to find an association between a higher number of chronic conditions and higher healthcare costs
  - Previous research has found that the average annual out-of-pocket cost for patients with 2 chronic medical conditions exceeds $10,000
2. You talk with the patient about the cost of care for chronic medical conditions. In the current study by Meraya and colleagues, which of the following combinations of chronic illness was the ***least*** expensive in the cost of annual care?
  - Arthritis/heart disease
  - Diabetes/hypertension
  - Arthritis/diabetes
  - Hypertension/heart disease
3. Four months later, the patient is admitted to the hospital for a non-ST-segment elevation myocardial infarction. She is treated successfully but is now even more worried about the costs of her care. Which of the following statements regarding adults with all 4 chronic illnesses evaluated in the current study is ***most*** accurate?
  - Heart disease did not substantially alter the cost of care among patients with diabetes
  - Heart disease did not substantially alter the cost of care among patients with hypertension
  - The presence of all 4 conditions was only marginally more expensive compared with the presence of diabetes and heart disease only
  - The presence of all 4 conditions was not associated with higher healthcare costs compared with 3 diagnoses of chronic illness
4. According to the results of the current study, what can you tell this patient now about her out-of-pocket costs?
  - There were no statistically significant differences in out-of-pocket costs based on different combinations of chronic disease
  - The presence of arthritis significantly increased out-of-pocket costs
  - The presence of heart disease significantly increased out-of-pocket costs
  - Higher numbers of chronic illnesses were associated with increased out-of-pocket costs

**Evaluation**
